# Photosynthetic epibionts and endobionts of Pacific oyster shells from oyster reefs in rocky versus mudflat shores

**DOI:** 10.1371/journal.pone.0185187

**Published:** 2017-09-21

**Authors:** Laurent Barillé, Anthony Le Bris, Vona Méléder, Patrick Launeau, Marc Robin, Ioanna Louvrou, Lourenço Ribeiro

**Affiliations:** 1 Laboratoire Mer Molécules Santé (EA2160), Institut Universitaire Mer et Littoral (FR_C 3473), Université de Nantes, Nantes, France; 2 LETG-Geolittomer (UMR 6554, CNRS), Institut Universitaire Mer et Littoral (FR_C 3473), Université de Nantes, Nantes, France; 3 Laboratoire de Planétologie et Géodynamique (UMR 6112, CNRS), Institut Universitaire Mer et Littoral (FR_C 3473), Université de Nantes, Nantes, France; 4 Department of Ecology and Systematics, Faculty of Biology, Panepistimiopolis, University of Athens, Athens, Greece; 5 Marine and Environmental Sciences Centre, Faculdade de Ciências, Universidade de Lisboa, Lisbon, Portugal; Institute of Oceanology, Chinese Academy of Sciences, CHINA

## Abstract

The Pacific oyster, *Crassostrea gigas* (Thunberg), is the main bivalve species cultivated in the world. With global warming enabling its reproduction and larval survival at higher latitudes, this species is now recognized as invasive and creates wild oyster reefs globally. In this study, the spatial distribution of photosynthetic assemblages colonizing the shells of wild *C*. *gigas* was investigated on both a large scale (two contrasting types of reefs found in mudflats and rocky areas) and a small scale (within individual shells) using a hyperspectral imager. The microspatial distribution of all phototrophs was obtained by mapping the Normalized Difference Vegetation Index (NDVI). Second derivative (δδ) analyses of hyperspectral images at 462, 524, 571 and 647 nm were subsequently applied to map diatoms, cyanobacteria, rhodophytes and chlorophytes, respectively. A concomitant pigment analysis was carried out by high performance liquid chromatography and completed by taxonomic observations. This study showed that there was high microalgal diversity associated with wild oyster shells and that there were differences in the structure of the phototropic assemblages depending on the type of reef. Namely, vertically-growing oysters in mudflat areas had a higher biomass of epizoic diatoms (hyperspectral proxy at δδ_462_ nm) and were mainly colonized by species of the genera *Navicula*, *Nitzschia* and *Hippodonta*, which are epipelic or motile epipsammic. The assemblages on the horizontal oysters contained more tychoplanktonic diatoms (*e*.*g*. *Thalassiosira pseudonana*, *T*. *proschkinae* and *Plagiogrammopsis vanheurckii)*. Three species of boring cyanobacteria were observed for both types of reef: *Mastigocoleus testarum*, *Leptolyngbya terrebrans*, and *Hyella caespistosa*, but the second derivative analysis at 524 nm showed a significantly higher biomass for the horizontally-growing oysters. There was no biomass difference for the boring chlorophyte assemblages (δδ_647_ nm), with two species: *Eugomontia testarum* and *Ostreobium quekettii* observed for both types of reef. This study shows that oyster shells are an idiosyncratic but ubiquitous habitat for phototrophic assemblages. The contribution of these assemblages in terms of biomass and production to the functioning of coastal areas, and particularly to shellfish ecosystems, remains to be evaluated.

## Introduction

Pacific oyster reefs are a growing habitat in temperate coastal areas, spreading in Europe and America, with a polarward expansion [1]. The species *Crassostrea gigas* was introduced worldwide for aquaculture following overexploitation of native populations. As a consequence of global warming, cultivated oysters began to reproduce at higher latitudes, with increasingly successful larval settlement leading to the development of these biogenic reefs [2]. These reefs are mainly known for the clusters of vertically-growing oysters, particularly in soft-bottom environments such as tidal flats where they create three-dimensional hard-substrate structures [3]. However, oysters can also colonize large rocky areas where they grow horizontally, forming a single layer tightly adhering to the substrate [4]. The structure of the habitat is therefore diverse, as are the shells themselves, characterized by variations in surface roughness, color, and sediment deposition. Microspatial variations influence the nature of the biota colonizing hard surfaces [5], and oysters shells have long been known to host a large diversity of organisms [6]. Most studies, however, have focused on colonization by metazoa and macrophytes [7,8] and less attention has been paid to microalgae and cyanobacteria.

Epibiosis is a widespread phenomenon in the marine environment [9]. According to Walker and Miller [10], the organisms that infest the surfaces of organic substrates are referred to as epibionts while those that live mostly or wholly within the tissues or body parts of other living or dead organisms (basibionts) as endobionts. The body surface of many metazoans is colonized by epibionts, including microepibionts such as bacteria, microalga, protozoa [11]. Boring communities are also prominent features, colonizing a variety of hard substrates not only of inorganic origin (limestones, dolostones, ooliths) but also calcified parts of organisms (skeletons or thalli) such as mollusk shells, calcareous red algae, coral reefs, bones, foraminifera (*e*.*g*. [12–15]). Mollusk shells are ubiquitous in coastal areas and provide abundant habitats whose importance and functional role have been overlooked [16]. Within the Mollusca phylum, bivalve and gastropod shells host photosynthetic communities composed of cyanobacteria, diatoms, chlorophytes and rhodophytes [17–21]. An early description of the microflora colonizing oyster shells reported the presence of cyanobacteria and chlorophytes [22]. Diatoms and spores/propagules of rhodophytes were later observed as significant component of oyster microepibiont phototrophic assemblages [6,7,23]. In most studies dealing with microepibionts, quantification is an issue and the analysis of the spatial distribution at microscale has seldom been addressed. In fact, the phototrophic communities found on mollusk shells share many similarities with epilithic microalgae found in rocky areas. There are constraints with the conventional sampling techniques based on the removal of rock surfaces [24], which are not adapted to studying the microspatial distribution characterizing epilithic biofilms. This promoted the emergence of remote-sensing techniques at visible near-infrared (VNIR) wavelengths to analyze these biofilms at a high spatial resolution with a non-invasive approach [25].

Non-intrusive analytical techniques based on the spectral properties of phototrophic assemblages have been increasingly used to describe marine biofilms [26,27]. The spectral reflectance (ratio of upwelling radiance and downwelling irradiance) of microalgal assemblages at VNIR wavelengths is essentially related to the phytopigment composition, abundance, and substratum contribution [28,29]. Most of these studies used field-spectroradiometers with a high spectral resolution (more than a hundred spectral bands), which can resolve subtle phytopigment absorption bands, but did not provide any spatial information. Some authors [25,30] tested various VNIR imaging systems with a high spatial resolution to map intertidal epilithic microalgae, but the low spectral resolution of the different sensors prevented the mapping of the diversity of the main microalgal classes. This can be overcome by imaging hyperspectral cameras characterized by a high spectral and spatial resolution [31]. This technique has the potential for innovative applications in ecology, such as the spectral camouflage of crabs [32], or the microspatial variability of calcified macroalgae epiphytes [33]. Hyperspectral imaging technology should find wide applications in the study of photosynthetic microepibionts but, to our knowledge, this is the first time it has been applied to map oyster shells at microscale.

The present study investigated spatial variations in the structure of the phototrophic assemblages growing on the shells of host oysters, *Crassostrea gigas*, using hyperspectral imagery. The spatial distribution of photosynthetic assemblages colonizing wild oyster shells was investigated on both a large scale (two contrasting types of reefs found in mudflats and rocky areas) and a small scale (within individual shells). We quantified the epibiont and endobiont assemblages using vegetation indices commonly used in remote-sensing to monitor the biophysical and biochemical properties of vegetation. A concomitant pigment analysis of these photosynthetic assemblages was carried out by high performance liquid chromatography and completed by taxonomic observations. We hypothesized that the structure of these assemblages (biomass, diversity) should differ between the two types of reefs and that microscale hyperspectral imaging is an appropriated technique to detect these differences.

## Materials and methods

**Fig 1:**
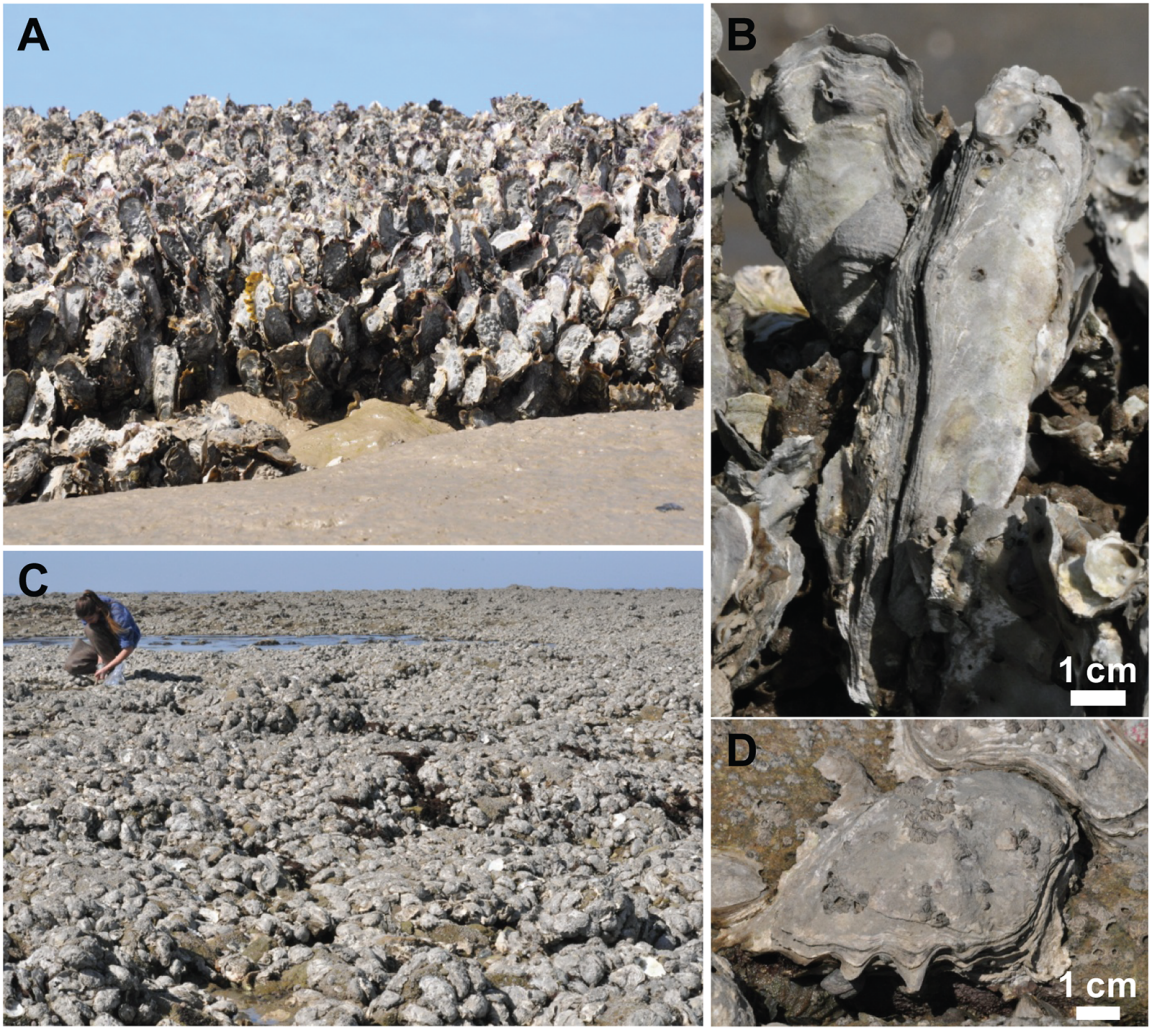
Typology of *Crassostrea gigas* wild oyster reefs. (A) Clusters of vertical oysters surrounded by mudflats; (B) Details of vertically-growing oysters; (C) Horizontal colonization of large rocky areas; (D) Details of a horizontally-growing oyster; the shell is colonized by a few cirripeds.

Wild oyster reefs were sampled in Bourgneuf Bay, located south of the Loire estuary on the French Atlantic coast (47°02’ N, 2°07’ W). In this macrotidal bay with a maximum tidal amplitude of 6 m, 100 km^2^ of the total bay area (340 km^2^) is intertidal with large mudflats [34]. It is characterized by highly turbid waters associated with the resuspension of soft-bottom sediments. The annual mean concentration of suspended particulate matter is of the order of 150 mg.L^-1^ with extreme values >1 g.L^-1^ during spring tides [35]. The Pacific oyster *Crassostrea gigas* (Thunberg), has been cultivated there since its massive importation starting in 1972, to replace the Portuguese oyster *Crassostrea angulata* decimated by a viral disease [36]. This bay was considered the northern boundary of *C*. *gigas* expansion at the time of its introduction into Europe [37]. Two distinct forms of oyster reefs were observed: clusters of vertical oysters found in rocky spots within a mudflat, building three-dimensional dense reefs in the muddy area (Fig 1A and 1B) and oysters growing horizontally creating large flat reef structures in the rocky areas (Fig 1C and 1D). In the muddy area, oyster shells were dark and partially covered by mud, while in rocky areas, there was a lower sediment deposition and oyster shells had a brighter color. One hundred oysters were sampled (50 from each reef type) and brought back to the lab in a cooler for hyperspectral, chromatographic and taxonomic analyses. Only the flat upper valves were kept and analyzed. We calculated the average surface of oyster shells to check if surface variations should be taken into account. All valves were visually free of any macrophyte vegetation but were often colonized by barnacles (*Chthamalus* spp. and *Elminius modestus*) which were the main epibiotic macrofauna. Sediment particles were often deposited on the shells, particularly for the vertical oyster reef. Bright white shells from dead oysters washed onto the shore were also collected and processed to obtain a spectral reference devoid of any type of biocolonization. There was no specific permissions required to sample oysters in the study site which belong to the public maritime domain. The field study did not involve endangered or protected species.

### Hyperspectral image analysis

Images were acquired with a HySpex camera set up in the laboratory. The HySpex VNIR 160 camera has a spectral resolution of 4.5 nm and a spectral sampling of 3.7 nm in 160 contiguous channels between 400 and 950 nm. The camera was fixed at 1 m above the samples to obtain square pixels with a spatial resolution of around 200 μm. Samples were isolated from the ambient light and the artificial illumination was controlled by two halogen quartz lamps (100 W). The optimal integration time was 20 ms to improve the signal-to-noise ratio. Reflectance was first determined by measuring the ratio between the light reflected from a calibrated 20% gray reference panel (Spectralon) and the light reflected by oyster shells. Reflectance was calculated by dividing each pixel of the image by the mean intensity of Spectralon in the 400–950 nm wavelength range. Minimum Noise Fraction (MNF) transformations combined with a band pass filter of 9 nm were applied to images to remove noise and redundant information. Polygon layers were applied to individualize and extract the pixels corresponding to each oyster. A continuum removal process was applied to eliminate background influences due to structural variations (color, microrelief) of the shell itself and to retrieve absorption features that are specific to photosynthetic and accessory pigments [38]. The upper envelope of spectra (= continuum) was modeled by straight lines that are tangential to local maxima of reflectance in the NIR at 750–850 nm. Each spectrum was then divided by its corresponding straight line.

### Spectral analysis

Ratio and hyperspectral (derivative analysis) vegetation indices commonly used in remote sensing have been applied to map phototrophic epibionts [28]. The NDVI (Normalized Difference Vegetation Index) was first calculated using the chlorophyll *a* absorption band at 673 nm. Since this pigment is ubiquitous among all photosynthetic organisms, this index was used to analyze the spatial distribution of the whole community of photosynthetic epibionts: NDVI = (R_750_ –R_673)_ / (R_750_ + R_673_), where R_750_ is the reflectance at 750 nm in the near infrared (NIR) and R_673_ is the red reflectance at 673 nm. A lower threshold at 0.05 was applied to exclude shell pixels without photosynthetic organisms but which displayed a positive NDVI. This value was chosen because it corresponded to the maximum NDVI found for the shells with no biocolonization. However, this index could not identify the main classes of photosynthetic organisms that colonize oyster shells. The analysis of the literature cited in the introduction gave us *a priori* clues about the classes of photosynthetic organisms that could be expected on Mollusk shells, either epilithic or endolithic: diatoms, chlorophytes, cyanobacteria and rhodophytes, the latter in the form of spores and propagules. Second derivative (δδ) were therefore calculated for each image and second derivatives peaks were used to identify these main classes: δδ_524_ and δδ_647_ for cyanobacteria and chlorophytes, respectively [39], δδ_571_ for rhodophytes [29] and we identified δδ_462_ for diatoms. Positive second derivative values at the four diagnosis wavelengths were used to attribute each pixel to a group, but the same pixel could be attributed to multiple groups if more than one wavelength had positive values [33]. The second derivative spectra of dead oyster shells which did not show any peak at the wavelengths corresponding to the absorption bands of the different pigments, were used as a control.

### High performance liquid chromatography (HPLC) analysis

After radiometric measurements, the imaged shells (24 for each reef type) were immediately frozen at -80°C and lyophilized for 72 hours for pigment extraction. Then the two types of shell (horizontal *vs*. vertical) were entirely crushed into a powder and subjected to the same extraction protocol which was carried out in the dark with 5 to 15 mL of 95% cold buffered methanol (2% ammonium acetate) for 15 min at -20°C. After centrifugation (at 3000 g, 15 min, 4°C), the supernatant was filtered using a Whatman membrane filter (0.2 μm) and diluted volume-to-volume in 1M ammonium acetate. A volume of 100 μL was injected for 30 min in a Waters SunFire C18 column (4.6 mm x 150 mm; 3.5 μm particle size) preceded by a pre-column. The elution solvents used were 1M of ammonium acetate in methanol (20:80) and methanol-acetone (60:40). The solvent gradient adapted by [40] was a flow rate of 1 mL.min^-1^. Pigment extracts were analyzed using their elution times and their absorption characteristics measured by a photodiode array at 440 nm and a fluorescence detector. Peaks were calibrated with standards from Sigma and DHI (DHI, Hørsholm, Denmark), but the HPLC protocol was not suitable for hydrosoluble pigments (*e*.*g*.phycocyanin and phycoerythrin) characteristic of rhodophytes. HPLC data were qualitatively used here to identify the main classes of photosynthetic microorganisms and help in the identification of the second derivative peaks. However, the concentration of fucoxanthin was quantified to analyse the relationship with the second derivative at 462 nm.

### Taxonomic identification

#### True-boring endobionts

Shell-boring endobionts were identified with a Carl Zeiss Axiostar Plus light microscope and pictures were taken of preserved shell samples (in 4% formaldehyde solution) using a Sony Cybershot DSC-F717 digital camera. The epibiotic macrofauna was removed and shell fragments were dissolved using Pereny’s solution (10% HNO_3_, 0.5% Cr_2_O_3_, 95% C_2_H_5_OH in proportion 4:3:3; [22]). The extracted euendoliths were observed on glass slides from 10 randomly chosen vertical and horizontal oysters.

#### Photosynthetic epibionts

The remaining shells were brushed and washed individually but in order to recover enough material for the analysis, all the sediment collected with 20 horizontal oysters was pooled and the same was done for 20 vertical oysters. Samples were kept in disposable polypropylene tubes to which was added 1 mL of a 2.5% glutaraldehyde solution and were stored at 4°C for later processing. Cells were extracted from the sediment using an isopycnic separation technique with silica sol Ludox HS-40 (Sigma-Aldrich, USA) that separates the organic material from mineral particles [41]. Preliminary observations revealed that diatoms dominated the epilithic assemblages. Samples were carefully oxidized by hydrogen peroxide (30%) at 90°C. Permanent slides were made from the cleaned diatom material mounted in Naphrax (Northern Biological Supplies Ltd., Ipswich, UK). Diatom identification and cell counts were made in 50 randomized ocular fields using a 100x oil immersion objective of a Zeiss Axioskop 50 microscope, equipped with differential interference contrast optical microscopy. More than 400 frustules/valves were counted, with the abundance of each taxon expressed as its relative percentage. Diatom identification mostly followed [41,42] and references therein. Relative abundances of diatom taxa were also allocated to four size classes, which comprised the very small (<100 μm^3^), small (100–250 μm^3^), medium-sized (250–1000 μm^3^) and large (>1000 μm^3^) diatoms (cf. [43]). Cell biovolume was either directly obtained from previous works [43,44] or calculated from biometric measurements made during the LM observations following [45]. A growth-form was attributed to each individual taxon following literature search on the auto-ecology of the species and genera. Three growth forms were considered attached to the substrate (*i*.*e*. oyster shells), namely: adnate, tube-dwelling and stalked diatoms, whereas four other growth forms were considered non-attached or free-living: epipelic, motile epipsammic, planktonic and tychoplanktonic diatoms. Species richness (S = number of species) and Shannon diversity index (H') were calculated to describe the assemblages.

### Statistical analysis

The normality and heteroscedasticity of data distributions were checked before each analysis using the Shapiro-Wilk test. Student t-tests were used to test the null hypothesis that there were no differences between mean NDVI and shell surfaces between horizontal and vertical oysters. F-test of equality of variance was used to test the hypothesis that NDVI variability was the same between the two types of oyster shell. The comparison between second derivative values was tested with PERMANOVA. The null hypothesis stated that there were no differences between colonized and dead oyster shells used as a control. The alternative hypothesis postulated that they were significant differences with dead oyster shells. *A posteriori* pairwise comparisons between the two types of oyster shell was then run to test if they were significant differences at four wavelengths (δδ_462_, δδ_524_, δδ_571_, δδ_647_). Spearman correlations were calculated between second derivatives to test the relevance of δδ_462_ as a proxy to identify diatoms. All statistical analysis were performed with PAST [46].

### Ethics statement and human subjects

The individual in Fig 1C of this manuscript has given written informed consent (as outlined in PLOS consent form) to publish these case details.

## Results

### Spectral reflectance and vegetation index

**Fig 2:**
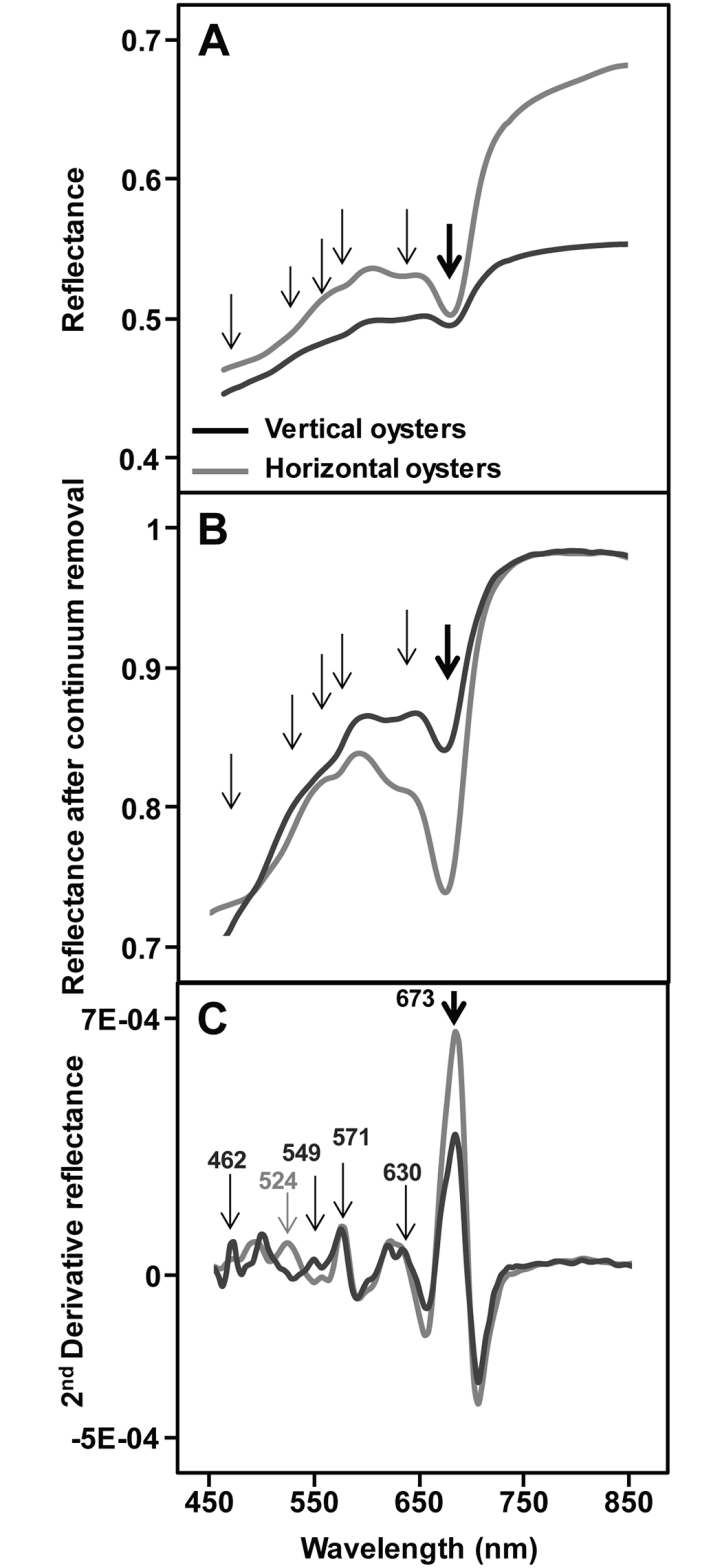
Example of spectral signatures of vertically- and horizontally-growing oysters obtained with the HySpex imaging spectrometer. (A) Reflectance spectra; the thick arrow indicates the chlorophyll *a* 673 nm absorption band. The thin arrows indicate other absorption features, sometimes corresponding to slope variations. (B) Removed-continuum spectra (see Material & methods). (C) Second derivative spectra with the main peaks associated with diagnostic wavelengths: 462, 549, and 630 nm for diatoms, 524 nm for cyanobacteria, 571 nm for rhodophytes (see Material & methods).

**Fig 3:**
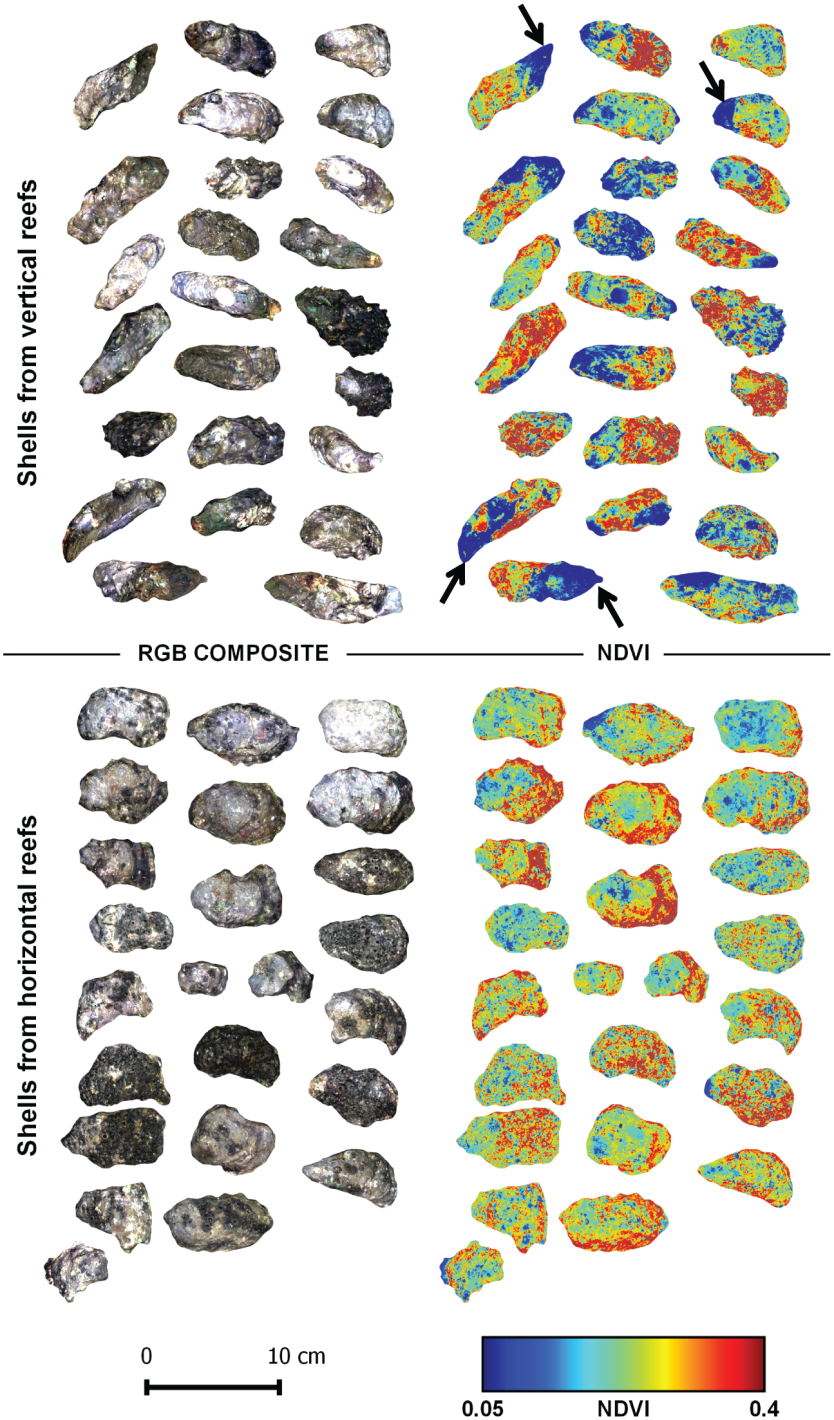
RGB composite color images (left) and corresponding NDVI spatial distribution images (right) of Pacific oyster shells sampled in two contrasting oyster reefs. Arrows indicate low NDVI areas close to the shell umbo.

The spectral shapes of the two types of oyster were similar overall in the VNIR wavelength range, but the darker vertical oyster shells always showed a lower albedo. A marked absorption band at 673 nm, characteristic of chlorophyll-*a* was systematically observed on the shells (Fig 2A, thick arrow). Since no macroalgae were visible at the surface of oyster shells whatever their type (vertical. *vs*. horizontal), this 673 nm absorption band was associated with microscopic organisms that could only just be detected visually for some shells by a greenish or light purple coloration of the shell. Other absorptions could be observed between 450 and 650 nm, some corresponding to slope changes in the reflectance spectra (Fig 2A, thin arrows). After the retrieval of their continuum, vertical and horizontal oyster reflectance spectra presented a similar shape in the near infrared (Fig 2B), but differences remained in the visible range. Second derivative spectra enhanced the changes in the reflectance spectra and were used to identify pigment absorptions (Fig 2C). The high resolution imaging of vertical and horizontal oyster shells confirmed the systematic presence of photosynthetic organisms with NDVI values ranging from 0.05 to 0.4 (Fig 3). The mean NDVI of vertical oyster shells was significantly higher than that of horizontal oyster shells (t-test, t = -2.47, *p*-value = 0.01; df = 48). The coefficient of variation was also higher for vertical oysters: 0.15 *vs*. 0.11 for horizontal oysters, and there was a significant difference between the variances of the two series (Fisher-test, F = 0.47, *p*-value = 0.03, df = 24). Vertical oysters were characterized by a contrasting NDVI distribution with areas of low NDVI values often located next to the shell umbo, and corresponding to zones where oysters were attached to another within a cluster (Fig 3, arrows). Horizontal oysters displayed a less heterogeneous NDVI distribution. There was no statistical difference between the average surface of horizontal (22.5 cm^2^) and vertical (22.3 cm^2^) oysters (t-test, t = -0.17, p-value = 0.86, df = 48).

### Pigment analysis and second derivatives

The diversity of lipophilic pigments detected by HPLC was common to both types of shell (vertical *vs*. horizontal) (Table 1). The simultaneous detection of different pigments could be used to identify the main algal classes. Fucoxanthin and chlorophyll *c* were marker pigments for diatoms. Neoxanthin, violaxanthin, and siphonaxanthin indicated the presence of chlorophytes (green algae).

**Table 1:** Pigment composition detected on oyster shells by HPLC. Pigment in bold represents pigments that were exclusive to taxonomic groups. Source: Jeffrey et al., [5,7].

Retention Time (min)	Peak Name	Algal division
3.4	Chlorophyllide *b*	Degradation products of chlorophyll *b*
4.8	Chlorophyllide *a*	Degradation products of chlorophyll *a*
6.1	**Chlorophyll *c***	Diatoms
6.9	Siphonaxanthin	Chlorophytes
7.8	**Fucoxanthin**	Diatoms
8.2	Neoxanthin	Chlorophytes
8.6	Violaxanthin	Chlorophytes
9	**Myxoxanthophyll**	Cyanobacteria
9.7	Zeaxanthin	Rhodophytes Cyanobacteria Chlorophytes
10.4	Lutein	Chlorophytes
11.7	Canthaxanthin	Chlorophytes Cyanobacteria
13.3	**Chlorophyll *b***	Chlorophytes
14.7	Chlorophyll *a*	All photosynthetic algae
20.6	*β*,*β*-caroten	Chlorophytes, Diatoms, Rhodophytes

**Fig 4:**
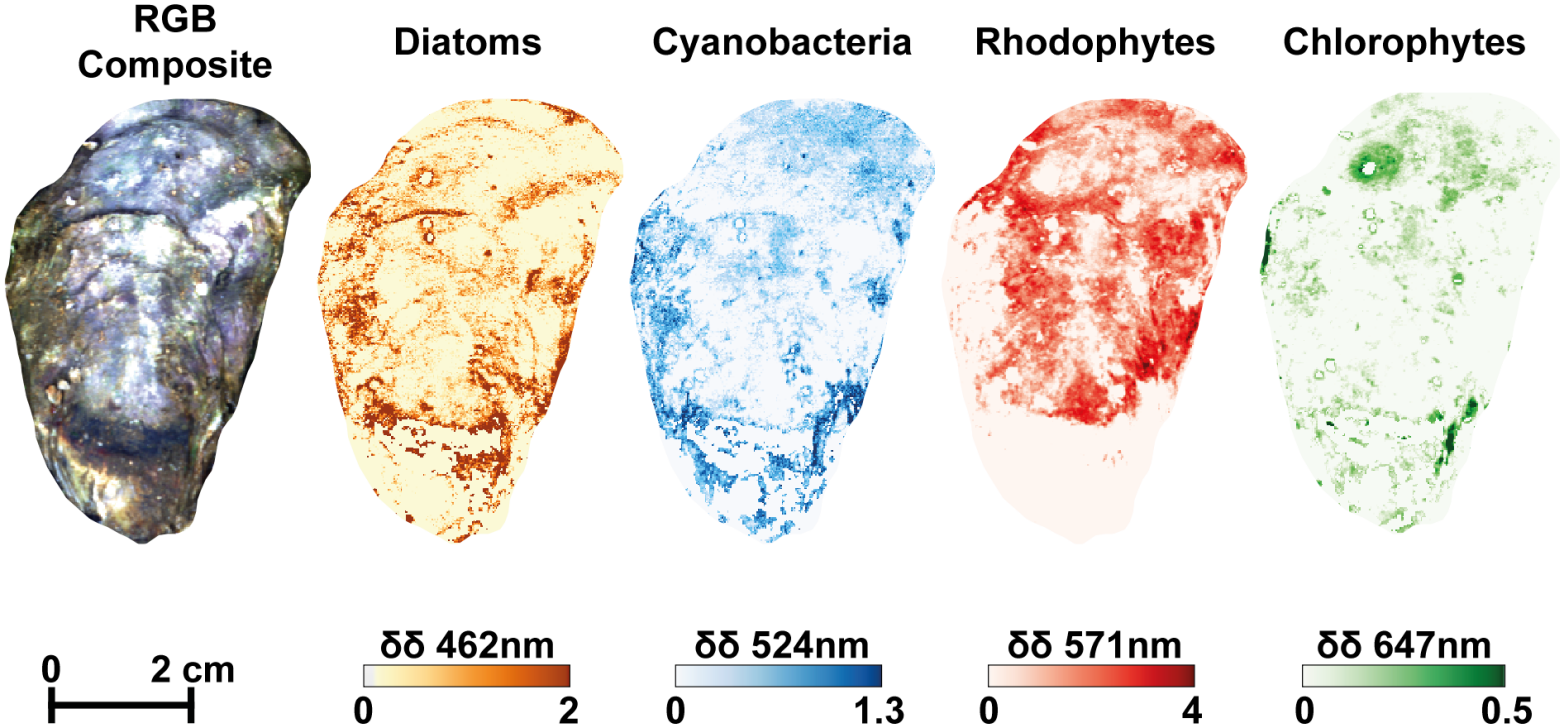
RGB composite color image of a vertical oyster shell (left) and the corresponding four second derivative images. Diagnostic wavelengths: 462 nm for diatoms, 524 nm for cyanobacteria, 571 nm for rhodophytes, 647 nm for chlorophytes. Second derivate values are multiplied by 10^4^.

**Fig 5:**
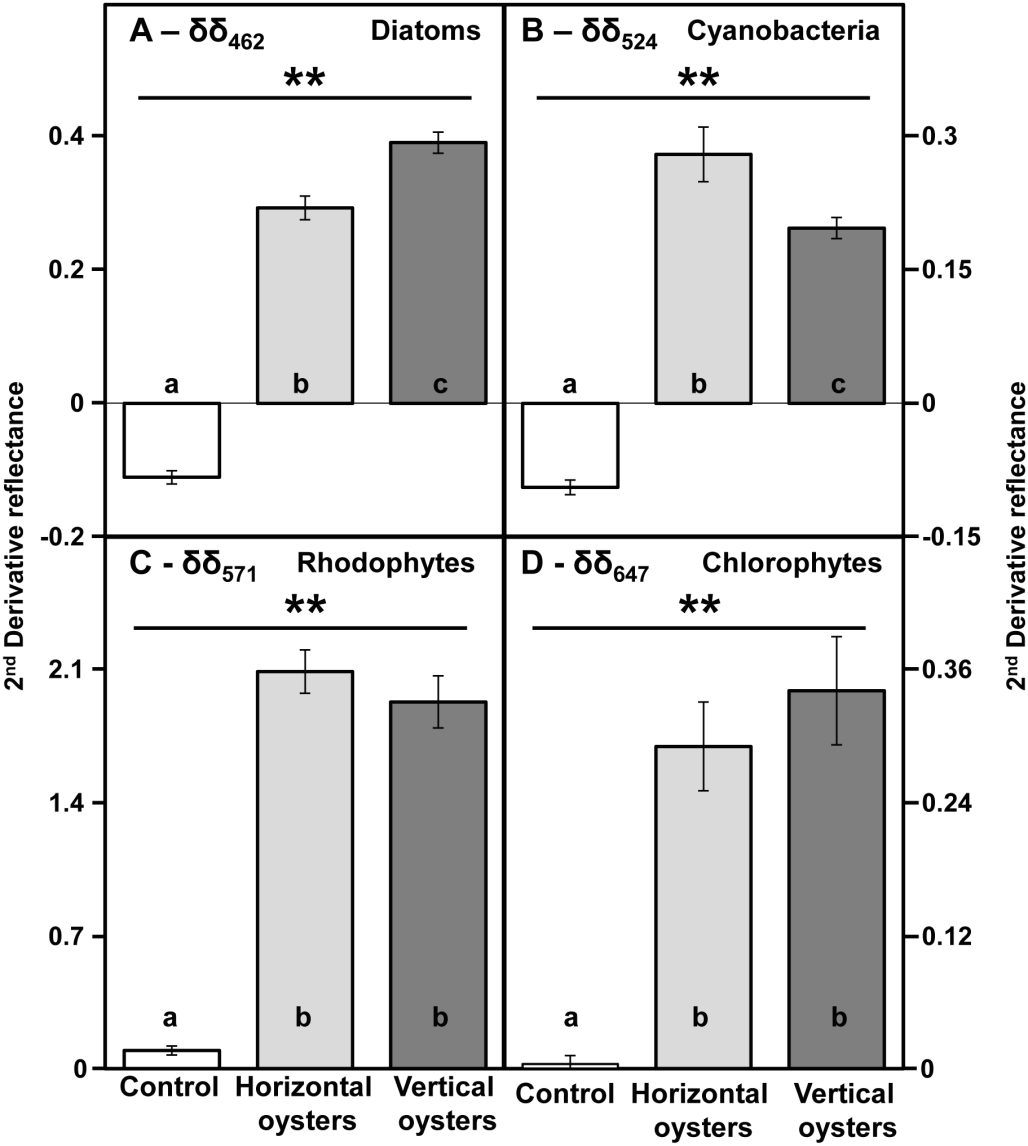
Comparison of mean second derivatives between vertically-growing (dark grey) and horizontally-growing (light grey) oysters. Diagnostic wavelengths: 462 nm for diatoms, 524 nm for cyanobacteria, 571 nm for rhodophytes, 647 nm for chlorophytes. Bright white shells from dead oysters washed onto the shore were used as a control with no biocolonisation. Means are presented with their confidence intervals at 95%. The double asterisk indicates a highly statistically significant difference (p<0.01) with the control. Within each graph, bars sharing the same letter are not significantly different (PERMANOVA, pairwise tests, p > 0.05).

Myxoxanthophyll and canthaxanthin indicated the presence of cyanobacteria. While some pigments were not specific to one class (*e*.*g*. zeaxanthin, *β*-carotene), fucoxanthin, myxoxanthophyll and chlorophyll *b* could be specifically associated with diatoms, cyanobacteria and chlorophytes respectively. These three groups were detected on both types of shell. Two of the biomarker pigments, myxoxanthophyll and chlorophyll *b*, could be identified on reflectance spectra by the second derivative values (δδ) corresponding to their main absorption bands at 524 nm and 647 nm, respectively. We used 462 nm to identify diatoms, rather than 549 nm, often used for fucoxanthin, since 462 nm was more discriminant in these shell mixed assemblages. The second derivative at 462 nm (δδ_462_) was indeed significantly correlated to δδ_549_ (r = 0.80, P<0.01, n = 48). Moreover, the relevance of 462 nm was also confirmed by a significant relationship between δδ_462_ and the concentration of fucoxanthin measured by HPLC (S1 Fig). For rhodophytes, there were no specific pigments identified by HPLC since the detected pigments zeaxanthin and *β*-carotene could belong to other classes. The second derivative at 571 nm was thus used as a spectral marker of the water-soluble pigment phycoerythrin common in red algae. For each oyster, distribution maps of the second derivatives at the four wavelengths (δδ_462_, δδ_524_, δδ_571_, δδ_647_) were obtained to estimate the distribution of the main photosynthetic organisms: diatoms, cyanobacteria, rhodophytes, and chlorophytes, respectively (Fig 4). Each group displayed visually heterogeneous spatial distributions without any obvious spatial pattern. The comparison between the mean derivative values calculated from the two series of high resolution images of oyster shells indicated that vertical and horizontal oysters were always significantly different from the control (Fig 5, PERMANOVA, *p*-value = 0.001, df = 71). Pairwise comparisons between vertical and horizontal oysters indicated significant differences for δδ_462_ (t = 4.62, *p*-value = 0.001) and δδ_524_ (t = 5.24, *p*-value = 0.001) (Fig 5A and 5B). However, there was no significant difference for δδ_571_ (test = 1.84, *p*-value = 0.073) and δδ_647_ (t = 1.64, *p*-value = 0.111) (Fig 5C and 5D). Diatoms showed a higher second derivative for vertical oysters but it was the opposite for cyanobacteria. There were no significant correlations between any second derivative for horizontal oysters, and only one significant correlation between δδ_462_ (diatoms) and δδ_524_ (cyanobacteria) for vertical oysters (r_*Pearson*_ = 0.44, P<0.05, n = 24).

### Taxonomic identification

#### Endobionts

**Fig 6:**
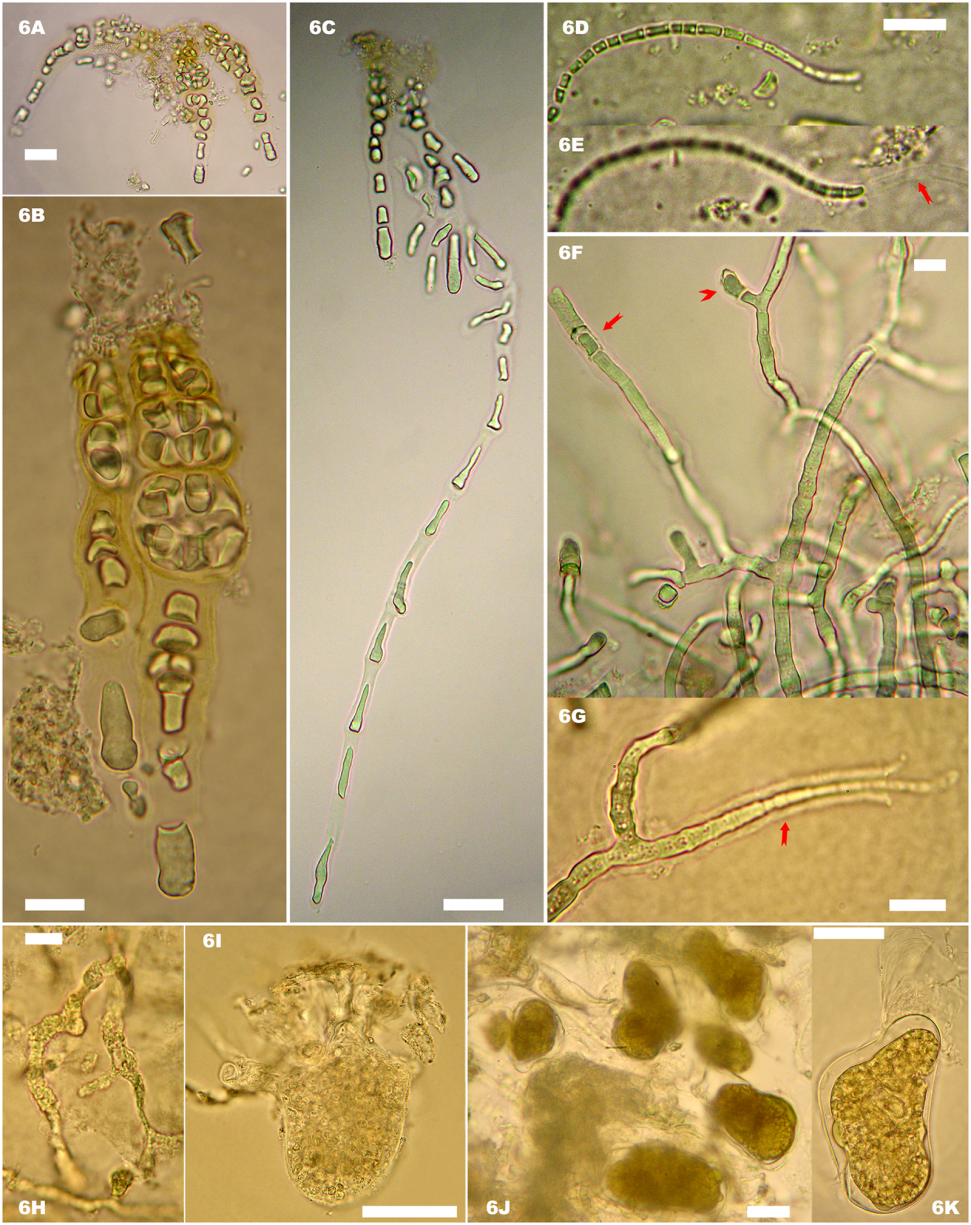
Photosynthetic euendoliths in host shells of *Crassostrea gigas*, light microscope. (A-F) Cyanobacteria. (A-C) *Hyella caespitosa*. (A) General view of the boring pattern near the surface of the oyster shell. (B) Short pseudofilaments. (C) Boring and almost perpendicular to the shell surface long pseudofilament. (D)Filament of *Leptolyngbya terebrans*. Note the trichome (D) and the empty mucilaginous sheath (E). (F-G) *Mastigocoleus testarum*. Note on (F) a short lateral T-branch bearing a terminal heterocyte (arrow head) and an infrequent intercalary heterocyte (arrow). (G) Multicellular 'hair-like' filament (arrow). (H-K) Chlorophytes. (H) Richly branched filaments of the siphonal *Ostreobium quekettii*. (I) Euendolithic *Codiolum* phase with multiple rhizoids of an undetermined ulotrichalean algae. (J-K) *Eugomontia sacculata*. (J) Endolithic, septate, branched, sporophyte filaments of *Eugomontia sacculata* with the formation of numerous large sporangial swellings. (K) Sporangium detached from the sporophyte filament of *E*. *sacculata*. Note the layered walls. Scale bars: 10 μm (B, D, E F, G, H); 20 μm (A, C, J, K); 50 μm (I).

Six taxa of true-boring endobionts were identified as colonizing within the oyster shells (Fig 6). There were three cyanobacteria: *Mastigocoleus testarum* Lagerheim (Hapalosiphonaceae, Nostocales), *Leptolyngbya terebrans* Bornet et Flahault (Leptolyngbyaceae, Synechococcales) and *Hyella caespitosa* Bornet et Flahault (Hyellaceae, Pleurocapsales) and three chlorophytes: *Ostreobium quekettii* Bornet et Flahault (Ostreobiaceae, Bryopsidales), *Eugomontia sacculata* Kornmann (Gomontiaceae, Ulotrichales) and a *Codiolum* phase of an undetermined ulotrichalean green alga. These six species of endobionts were observed for both types of shell except for the *Codiolum* phase which was only observed with horizontal oysters.

#### Epibionts

Epibionts were essentially dominated by diatoms. Both diatom assemblages had a total of 40 genus and 93 taxa (Table 2). The complete list of taxa identified is given in supporting information (S1 Table). The assemblage collected on the vertical oyster shells was composed of 83 taxa (H’ = 3.8) whereas 53 taxa were found on the horizontal oyster (H’ = 3.1).

**Table 2:** List of the diatom genus found in vertical and horizontal oysters, including details of their relative abundance (%), and their life-forms.

Taxa	Species	Horizontal shells	Vertical shells	Life-form
*Achnanthes*	2	0.5	2.1	Stalked
*Amphora*	4	3.6	5.4	Motile epipsammic
*Astartiella*	1	0.2	0.6	Motile epipsammic
*Berkeleya*	1	0	2.3	Tube-dwelling
*Biremis*	1	0	0.2	Adnate
*Caloneis*	2	0.5	0.6	Epipelic
*Catenula*	1	0	0.4	Adnate
*Cocconeis*	2	0.5	0.2	Adnate
*Cyclostephanos*	1	0	0.2	Plankton
*Cyclotella*	2	0.9	0.4	Plankton
*Cymatosira*	1	0.7	2.3	Tychoplankton
*Delphineis*	1	3.8	1	Stalked
*Dimeregramma*	2	0.2	0.4	Stalked
*Diploneis*	2	0	0.6	Epipelic
*Entomoneis*	1	0	0.2	Epipelic
*Eunotogramma*	1	4.3	1.2	Adnate
*Fallacia*	3	0	1	Motile epipsammic
*Fragilaria*	1	0	0.2	Stalked
*Gyrosigma*	4	0.7	1.4	Epipelic
*Halamphora*	1	0	0.4	Motile epipsammic
*Hippodonta*	1	1.6	**6.2**	Motile epipsammic
*Hyalodiscus*	1	0	0.2	Plankton
*Licmophora*	1	0	0.2	Stalked
*Luticola*	1	0	0.2	Epipelic
*Melosira*	1	0	0.4	Tychoplankton
*Minidiscus*	1	0.5	0	Plankton
*Navicula*	19	**25.3**	**41.4**	Epipelic
*Nitzschia*	9	9	**10.5**	Epipelic
*Odontella*	1	0	0.2	Tychoplankton
*Climaconeis*	1	0	0.2	Epipelic
*Opephora*	3	2.9	1.2	Stalked
*Paralia*	1	0.2	0.4	Tychoplankton
*Parlibellus*	1	0.5	0.2	Tube-dwelling
*Plagiogrammopsis*	2	**10.4**	5.6	Stalked
*Planothidium*	5	2	3.5	Adnate
*Psammodictyon*	1	0	0.8	Epipelic
*Rhaphoneis*	1	0.2	0.6	Stalked
*Surirella*	1	0	0.2	Epipelic
*Thalassiosira*	7	**31.2**	5.8	Plankton & Tychoplancton
*Tryblionella*	1	0.5	0.8	Epipelic

**Fig 7:**
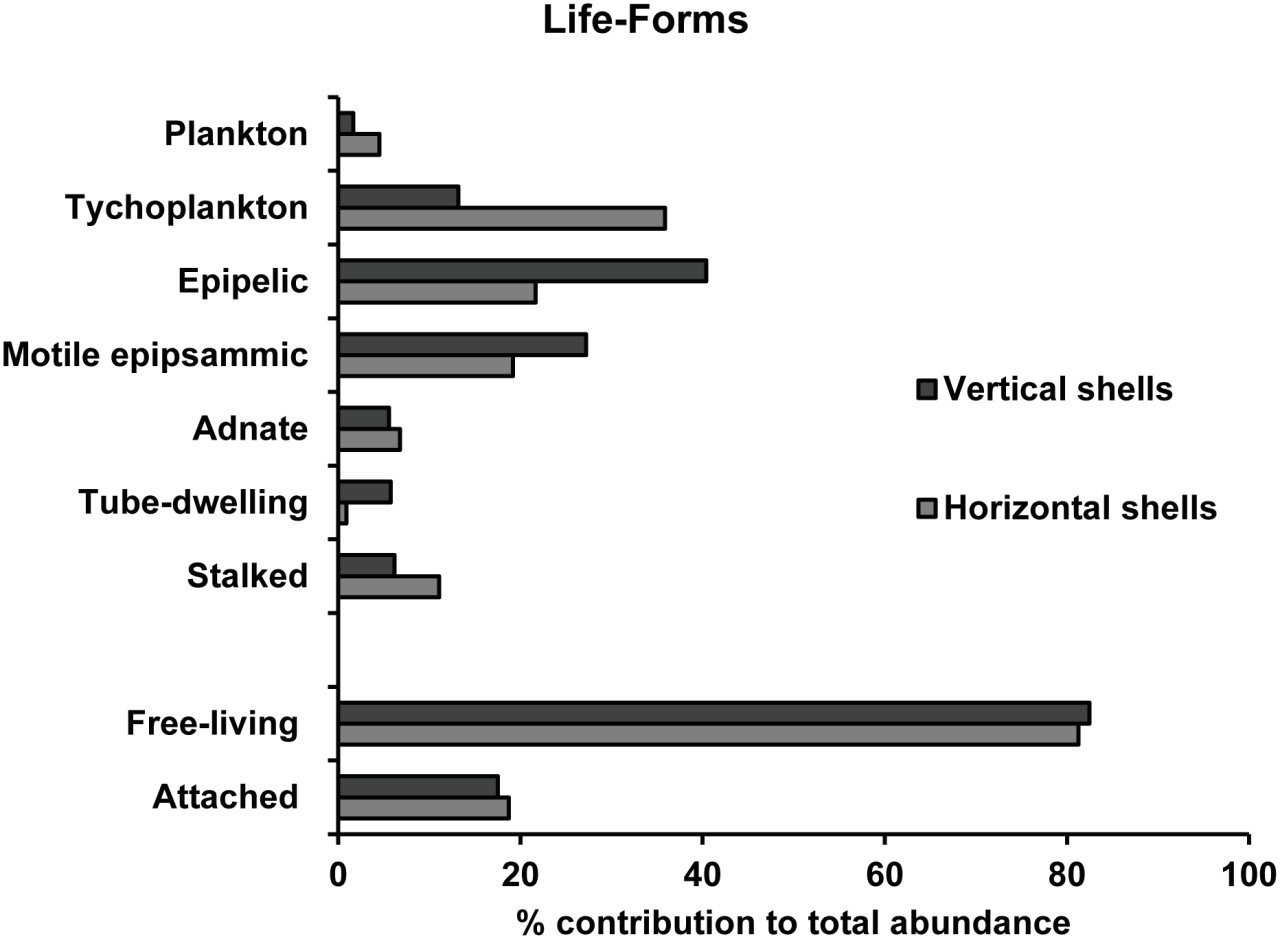
Percentage contribution of diatom life-forms to total diatom abundance found on oyster shells from two contrasting oyster reefs.

About 46% of the identified taxa were common to both assemblages and corresponded to 76% and 91% of cumulative abundances for vertical and horizontal oysters, respectively. The vertical oysters were mainly colonized by species of the genera *Navicula*, *Nitzschia* and *Hippodonta* (*e*.*g*. *Navicula recurva; N*. *diserta and Hippodonta caotica*), which are epipelic or motile epipsammic; whereas the assemblages in the horizontal oysters contained mostly tychoplanktonic diatoms (*e*.*g*. *Thalassiosira pseudonana*, *T*.*proschkinae* and *Plagiogrammopsis vanheurckii)* (S1 Table). With regard to growth forms, both assemblages had similar ratios of about 2 attached to 8 free-living diatoms, but there was a clear shift from epipelic diatoms (40% of relative abundance) in the vertical oyster assemblage to one dominated by tychoplankton (36%) in the horizontal oyster assemblage (Fig 7). In the latter assemblage, attached diatoms were mostly composed by stalked and adnate forms, whereas in the vertical oysters these diatoms were evenly distributed between the stalked, adnate and tube-dwelling forms. Whilst planktonic diatoms were slightly more abundant in the horizontal oysters, the converse occurred with the motile epipsammic growth form. Perhaps the clearest difference between the two assemblages concerned the size-class distribution. The assemblage in the horizontal oysters was dominated by small (100–250 μm^3^) and very small (<100 μm^3^) diatoms, corresponding to 36% and 43% of the cumulative abundances, respectively. In the vertical oyster, the assemblages were dominated by small (39%) and medium-sized diatoms (250–1000 μm^3^), which attained 32% of the cumulative relative abundance.

## Discussion

### An old story behind an intriguing spectral shape

Wild oyster shells originating from two types of reef (vertically-*vs*. horizontally-growing oysters) were imaged in this study with a high spectral (160 spectral bands in the VNIR) and spatial resolution (pixel of *ca*. 200 μm). Hyperspectral imagery has the advantage of providing rapid and non-invasive measurements compared to traditional sampling methods [31]. In this study, we used a laboratory equipment but there are portable imagers that can be used in the field or onboard vessels [31,32]. Compared to spectroradiometers [27,28,39] or fluorometric probes such as the BenthoTorch [47] that are instruments essentially measuring a single-point, hyperspectral imagers are able to resolve the spatial distribution of photosynthetic assemblages at a scale of millimeters. This can overcome the inherent spatial variability of benthic microalgal assemblages [31,47,48]. All shells, whatever the reef type, displayed an intriguing spectral shape with marked absorption features in the visible combined with a reflection in the NIR, which could be that of a soil or a rock. This type of spectral signature was first observed for a shellfish habitat of the eastern oyster *Crassostrea virginica* [49], using a high resolution field-spectroradiometer. These authors speculated about the presence of algal growth on the shells based on the characteristic chlorophyll *a* absorption at 675 nm without any further analysis of the nature of this vegetation. A similar observation was made for the shells of wild Pacific oysters *Crassostrea gigas* but the presence of photosynthetic microepibionts was suspected, since no visible macroalgae could be seen colonizing the shells [4]. Moreover, when these authors tried to map clusters of vertical oysters with an airborne hyperspectral sensor, significant confusion arose with pixels of microphytobenthos from the muddy surrounding areas. Microphytobenthic biofilms are generally dominated by diatoms, but they can also be composed of cyanobacteria, chlorophytes and euglenids [50]. This study confirmed that all oyster shells analyzed were colonized by photoautotrophs, since each individual shell was characterized by a positive NDVI value. Remote sensing of oyster reefs and intertidal shellfish habitats in general is a recent research field open to innovative approaches. For example, Synthetic Aperture Radar (SAR) exploiting microwaves has also been tested to map oyster and mussel beds in Europe [51] and South Korea [52] as a complement to VNIR wavelengths. To our knowledge our study is the first to analyze a mollusk shell reflectance at such a high resolution. Spectral resolution was important for selecting relevant wavelengths to identify assemblage composition, while high spatial resolution reduced the problem of patchiness and mixed pixels. The result obtained in this study will be useful for remote sensing at larger spatial scales, providing informations for building spectral libraries of wild oysters and improving the airborne hyperspectral mapping of oyster reefs [4]. However, behind these new data collected with a state-of-the-art hyperspectral imager, there lies an old story. In fact, the 19^th^-century scientists gave detailed descriptions of shell-boring microorganisms [22,53]. Bornet and Flahault [22] analyzed the shells of European oysters *Ostrea edulis* in a location just ten kilometers north of our study site and found several endolithic cyanobacteria and chlorophytes. Many scientists of the 20^th^ century completed these early works on shell-boring photosynthetic microorganisms [20]. Among them Schodduyn [6] who studied the epibionts colonizing the surface of European oyster shells found rhodophytes and diatoms. From these early works and in spite of different shell structures, two types of colonization, epizoic and shell-boring, and four classes of photosynthetic microepibionts: cyanobacteria, chlorophytes, diatoms and rhodophytes (spores and propagules) could *a priori* be expected on the shells of the Pacific oyster *Crassostrea gigas*.

### Diversity of photosynthetic epibionts

Spectral reflectance has been successfully used to determine the dominant taxonomic groups of microphytobenthos (*e*.*g*.[39,54,55]) but has not previously been applied to unravel the diversity of phototrophic epibionts. In fact, the use of a hyperspectral imaging system to map the spatial distribution of photosynthetic organisms at microscale is a recent technological application [56]. The derivative analysis of reflectance spectra enabled the separation of four absorption features, which were used to obtain spatial distribution maps and quantitative information about the four groups of photosynthetic epibionts and endobionts. Namely, δδ_462_, δδ_524_, δδ_571_, and δδ_647_ were used to map diatoms, cyanobacteria, rhodophytes and chlorophytes, respectively. Diatoms are generally identified by their absorption bands at ~550 nm for fucoxanthin [38,39] or at ~632 nm for chlorophyll *c* [40] but, in this study, there were overlaps with other absorptions and a more discriminant wavelength, less sensitive to taxonomic mixing, was chosen at 462 nm. Cyanobacteria were identified by the absorption band at 524 nm for myxoxanthophyll, rhodophytes at 571 nm for phycoerythrin, and chlorophytes at 647 nm for chlorophyll *b* [29,33,57]. There can be a shift of a few nm compared to the cited references, due to the different spectral resolution of the sensors. All oysters showed pixels with positive second derivative peaks at the four diagnostic wavelengths indicating the presence of the four groups of photosynthetic microorganisms on oyster shells. However, as illustrated in Fig 4, the microspatial distributions differed between each group. With the overall lack of correlation between the second derivative wavelengths, we speculated that the biomass/distribution of each group may not be related. It was beyond the scope of this work to quantify these spatial structures rigorously, but a further examination of second derivative hyperspectral images is a possibility. HPLC data were used to confirm the presence of the four groups of photosynthetic microorganisms and their diagnostic pigments. Oyster shells from the two types of reef shared a common pigment composition. All oysters had the pigment biomarkers of diatoms (chlorophyll *c* and fucoxanthin), cyanobacteria (canthaxanthin and myxoxanthophyll) and chlorophytes (chlorophyll *b* and neoxanthin) [57]. *β*-Carotene and zeaxanthin are common in rhodophytes, but are also present in the other groups. Zeaxanthin, more specific, can also be found in cyanobacteria and has led to an ambiguous diagnosis in mixed assemblages [39]. Phycoerythrin, not detected by the HPLC protocol used here, was probably responsible for the marked second derivative peak at 571 nm present in rhodophytes [33]. However, a caveat should be made here since this pigment can also be detected in cyanobacteria, with many benthic species being red [58]. Nevertheless, in this study it was associated with the characteristic rhodophyte reflectance spectrum, with its double-hump shape between 550 and 675 nm, as can be seen in *Porphyridium purpureum* [29]. A possible overestimation of the rhodophytes cannot, however, be excluded, particularly for mixed spectra. This would obviously be related to the presence of phycoerythrin in the endolithic cyanobacteria colonizing the shells. Raghukumar and colleagues [59] detected no phycoerythrin in cultures of *Leptolyngbya terebrans* colonizing various mollusk shells. Performing spectral and HPLC measurements on a pure endolithic cyanobacteria culture (*e*.*g*.;[60]), would certainly improve the remote-sensing approach developed in this work. The consistency of these spectral and pigment data was reinforced by taxonomic identifications. Three species of cyanobacteria were observed in the shell-boring assemblages (*Mastigocoleus testarum*, *Leptolyngbya terebrans* and *Hyella caespitosa*) as well as three species of chlorophytes (*Eugomontia sacculata*, *Ostreobium quekettii* and a *Codiolum* phase of an unidentified ulotrichalean alga). *Leptolyngbya terebrans*, *Eugomontia sacculata* and *Ostreobium quekettii* are cosmopolitan forms [20,61]. The former (syn. *Plectonema terebrans)* was the dominant shell-boring phototroph in *Crassostrea cucullata* [59]. It should be noted that the simple architecture of photosynthetic microborers, with their few available morphological diagnostic features, may lead to an underestimation of their diversity. Recent studies revealed *via* metabarcording the molecular diversity for microsiphonous taxa of the order Bryopsidales previously referred to as *Ostreobium* spp. [62,63]. A total of 93 taxa of epizoic diatoms were identified on wild *C*.*gigas* shells confirming the ubiquity and diversity of epizoic diatoms associated with the Mollusca phylum [18,19,21,64]. This diversity was related to the type of oyster reef, as shown in the next section.

### Difference between the two types of reef

At the macroscopic scale of a 1 m^*2*^ pixel, Le Bris et al. [4] showed that variations in reef geometry (three-dimensional in muddy areas *vs*. two-dimensional in rocky areas) and shell brightness (brighter when growing horizontally) were responsible for distinct spectral signatures. However, the main differences appeared in the NIR, and the more subtle changes occurring at visible wavelengths and involving photosynthetic and accessory pigments were not explored. Microspatial hyperspectral imaging revealed a striking difference in shell colonization. The biomass of these assemblages was estimated using a widely used remote-sensing proxy, the Normalized Difference Vegetation Index [28]. Results showed that vertically-growing oysters were characterized by assemblages with a higher biomass. Moreover, the high spectral resolution enabled a further analysis of which group of photosynthetic microorganisms was responsible for this biomass variation. The second derivative analysis has been successfully used to study microphytobenthos biofilms (*e*.*g*.[39,65]). We found that there were significant differences between the two types of reefs for δδ_462_ and δδ_524_, that are proxies for respectively diatom and cyanobacteria biomass. δδ_524_ was significantly higher in horizontally-growing oysters. Since the same three species were observed for the two reef types, this suggests that there was mainly a difference in biomass. δδ_462_ was significantly higher in vertically-growing oysters and the diatom assemblages had a more established and typical benthic community constituted by epipelic and epipsammic life-forms. Horizontal oysters were characterized by a higher fraction of tychoplanktonic species, and seemed to be more influenced by the suspension-resuspension cycles [43]. The epipelic diatoms of the vertical oyster assemblages were medium-sized (250–1000 μm^3^), while the *Thalassiosira* species in the horizontal ones were typically very small. These findings are consistent with the physical conditions of the locations of the two types of reef: in the rocky area where the oysters grow horizontally, there is a stronger hydrodynamism with less sediment deposited on the shells. On the contrary, vertical clusters of oysters are found near soft-bottom sediments, in areas of lower hydrodynamism. There, shells can be partially covered by muddy-sandy particles, which create a favorable substrate for epipelic and epipsammic species. The surprisingly high diversity of the two reef-type assemblages is probably the result of the increased substrate heterogeneity at the surface of oyster shells. Sediments deposited or trapped in shell interstices create soft-bottom microhabitats that can be exploited by residents from the nearby mudflats and sandflats. However, vertical oyster shells were also rougher and it cannot be excluded that these microspatial shell variations influenced the structure of the assemblages [5]. This would be consistent with the findings of D’alelio and colleagues [18] who showed that an increasing structural diversity in various gastropod shells promoted a greater complexity of the associated epizoic diatom assemblages. Gutiérrez and colleagues [16] pointed out that the provisioning of substrata was not the sole mechanism that may be responsible for an increase in species richness and we refer to their work for a comprehensive overview. Eventually, if one considers the living oyster itself, the host-microbiont interaction appears even more complex since mollusks may also provide nutrients through the products of their catabolism (biodeposits coated with mucus, dissolved excretion) which can be used by phototrophs [66,67].

## Conclusion

This study showed that high diversity of photosynthetic microorganisms was associated with wild oyster shells and that there were differences in the structure of the phototropic assemblages depending on the reef typology. Namely, vertically-growing oysters in mudflat areas had a higher biomass of epizoic diatoms. There was also a higher biomass of cyanobacteria on horizontally-growing oysters in rocky areas. There were no differences for chlorophytes and rhodophytes. For the latter, the results were only based on remote sensing data since there were no microscopic observations of spores, propagules and/or of the euendolithic *Conchocelis* phase, the sporophyte of the red macroalgae *Porphyra* [68], known to colonize oyster shells [69]. A future improvement in the remote-sensing approach would be to collect *Conchocelis* spectral signatures. The next ongoing step is to quantify the biomass of the epizoic and shell-boring assemblages using quantitative HPLC data coupled with hyperspectral data. This would help to provide a first estimation of the biomass of primary producers associated with wild oyster shells and assess their contribution at the level of an ecosystem.

## Supporting information

S1 Fig Relationship between fucoxanthin concentration (expressed in mass of pigment per oyster shell surface) and second derivative peak at 462 nm. Second derivate values are multiplied by 10^4^.
(PDF)

S1 Table Detailed list of all diatom taxa found in vertical and horizontal oysters, including details of their relative abundance (%), and their life-forms. (PDF)
