# Supplementary material for: Photosynthetic epibionts and endobionts of Pacific oyster shells from oyster reefs in rocky versus mudflat shores
Source: PLoS One. 2017 Sep 21;12(9):e0185187. doi: 10.1371/journal.pone.0185187 (PMC5608347; doi:10.1371/journal.pone.0185187)

SUPPORTING INFORMATION

S1 Figure. Relationship between fucoxanthin concentration (expressed in mass of pigment per oyster shell surface) and second derivative peak at 462 nm. Second derivative values are multiplied by 10^4.

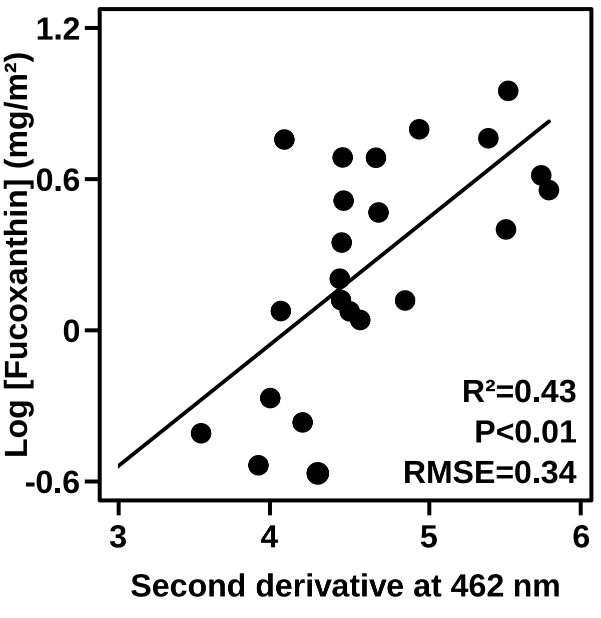

Supplement: S1 Fig — Second derivate values are multiplied by 104. (PDF) [file pone.0185187.s001.pdf]
