# Supplementary material for: Photosynthetic epibionts and endobionts of Pacific oyster shells from oyster reefs in rocky versus mudflat shores
Source: PLoS One. 2017 Sep 21;12(9):e0185187. doi: 10.1371/journal.pone.0185187 (PMC5608347; doi:10.1371/journal.pone.0185187)
Supplement: S1 Table — (PDF) [file pone.0185187.s002.pdf]

SUPPORTING INFORMATION

S1 Table. Detailed list of all diatom taxa found in vertical and horizontal oysters, including details of their relative abundance (%), and their life-forms.

	**Taxa**	**Vertical shells**	**Horizontal shells**	**Life-form**
#1	*Achnanthes longipes* C.Agardh	0.4	0.2	Stalked
#2	*Achnanthes parvula* Kützing	1.6	0.2	Stalked
#3	*Amphora* cf. *helenensis* Giffen	1.9	2.7	Motile epipsammic
#4	*Amphora* cf. *pediculus* (Kützing) Grunow ex A.Schmidt	1.6		Motile epipsammic
#5	*Amphora* cf. *subacutiuscula* F.R.Schoeman	0.6		Epipelic
#6	*Amphora* cf. *tenuissima* Hustedt	1.2	0.9	Motile epipsammic
#7	*Astartiella* cf. *bahusiensis* (Grunow) Witkowski, Lange-Bertalot & Metzeltin	0.6	0.2	Motile epipsammic
#8	*Berkeleya rutilans* (Trentepohl ex Roth) Grunow	2.3		Tube-dwelling
#9	*Biremis lucens* (Hustedt) K.Sabbe, A.Witkowski & W.Vyverman	0.2		Adnate
#10	*Caloneis* cf. *linearis* (Grunow) Boyer	0.2		Epipelic
#11	*Caloneis westii* (W.Smith) Hendey	0.4	0.5	Epipelic
#12	*Catenula adhaerens* (Mereschkowsky) Mereschkowsky	0.4		Adnate
#13	*Climaconeis inflexa* (Brébisson) Cox	0.2		Epipelic
#14	*Cocconeis guttata* Hustedt & Aleem	0.2		Adnate
#15	*Cocconeis scutellum* Ehrenberg		0.5	Adnate
#16	*Cyclostephanos dubius* (Hustedt) Round in Theriot et al.	0.2		Plankton
#17	*Cyclotella choctawhatcheeana* Prasad		0.7	Plankton
#18	*Cyclotella meneghiniana* Kützing	0.4	0.2	Plankton
#19	*Cymatosira belgica* Grunow	2.3	0.7	Tychoplankton
#20	*Delphineis minutissima* (Hustedt) Simonsen	1.0	3.8	Stalked
#21	*Dimeregramma minor* (Gregory) Ralfs ex Pritchard	0.2	0.2	Stalked
#22	*Dimeregramma minor* (Gregory) Ralfs ex Pritchard	0.2		Epipelic
#23	*Diploneis papula* (A.W.F.Schmidt) Cleve	0.2		Epipelic
#24	*Diploneis smithii* (Brébisson) Cleve	0.4		Epipelic
#25	*Entomoneis paludosa* (W.Smith) Reimer in Patrick & Reimer	0.2		Epipelic
#26	*Eunotogramma dubium* Hustedt	1.2	4.3	Adnate
#27	*Fallacia scaldensis* Sabbe & Muylaert	0.6		Motile epipsammic
#28	*Fallacia subforcipata* (Hustedt) D.G.Mann	0.2		Motile epipsammic
#29	*Fallacia tenera* (Hustedt) D.G.Mann	0.2		Epipelic
#30	*Fragilaria geocollegarum* Witkowski & Lange-Bertalot	0.2		Stalked

SUPPORTING INFORMATION

S1 Table (continued).

	Taxa	Vertical shells	Horizontal shells	Life-form
#31	*Gyrosigma acuminatum* var. *gallicum* (Grunow) Cleve	0.6		Epipelic
#32	*Gyrosigma* cf. *limosum* Sterrenburg & Underwood	0.6	0.5	Epipelic
#33	*Gyrosigma distortum* (W.Smith) Griffith & Henfrey	0.2		Epipelic
#34	*Gyrosigma littorale* (W.Smith) Griffith & Henfrey		0.2	Epipelic
#35	*Halamphora* cf. *abuensis* (Foged) Levkov	0.4		Motile epipsammic
#36	*Hippodonta caotica* Witkowski	6.2	1.6	Motile epipsammic
#37	*Hyalodiscus radiatus* (O'Meara) Grunow	0.2		Plankton
#38	*Licmophora gracilis* (Ehrenberg) Grunow	0.2		Stalked
#39	*Luticola mutica* (Kützing) D.G.Mann	0.2		Epipelic
#40	*Melosira nummuloides* C.Agardh	0.4		Tychoplankton
#41	*Minidiscus chilensis* Rivera		0.5	Plankton
#42	*Navicula agnita* Hustedt		0.2	Epipelic
#43	*Navicula aleksandrae* Lange-Bertalot, Bogaczewicz-Adamczak & Witkowski	1.0	0.2	Motile epipsammic
#44	*Navicula arenaria* Donkin	0.8	1.4	Epipelic
#45	*Navicula biskanterae* Hustedt	2.5		Motile epipsammic
#46	*Navicula* cf. *phyllepta* Kützing	3.3	0.7	Epipelic
#47	*Navicula* cf. *salinicola* Hustedt	3.9	5.0	Epipelic
#48	*Navicula* cf. *veneta* Kützing	0.2		Epipelic
#49	*Navicula diserta* Hustedt	7.2	9.7	Motile epipsammic
#50	*Navicula hamiltonii* Witkowski	0.2	0.2	Epipelic
#51	*Navicula perrhombus* Hustedt ex Simonsen	0.2		Epipelic
#52	*Navicula phyllepta* Kützing	0.8	0.7	Epipelic
#53	*Navicula ramosissima* (C.Agardh) Cleve	3.3	0.5	Tube-dwelling
#54	*Navicula recurva* (F.Meister) Witkowski	12.4	0.2	Epipelic
#55	*Navicula salinicola* Hustedt		4.5	Epipelic
#56	*Navicula sp.*1	1.9	0.5	Epipelic
#57	*Navicula sp.*2	0.2		Epipelic
#58	*Navicula spartinetensis* Sullivan & Reimer	0.6	0.5	Epipelic
#59	*Navicula subagnita* Proshkina-Lavrenko	1.2	1.1	Epipelic
#60	*Navicula viminoides* Giffen	1.6		Motile epipsammic
#60[a]	*Navicula spp.*	**41.4**	**25.3**	
#61	*Nitzschia* cf. *aequorea* Hustedt	1.4	1.6	Epipelic
#62	*Nitzschia* cf. *dubia* W.Smith	0.2	3.4	Epipelic
#63	*Nitzschia distans* W.Gregory	0.6		Epipelic
#64	*Nitzschia frustulum* (Kützing) Grunow	2.3		Epipelic

SUPPORTING INFORMATION

S1 Table (continued).

	Taxa	Vertical shells	Horizontal shells	Life-form
#65	*Nitzschia lorenziana* Grunow	0.2		Epipelic
#66	*Nitzschia pellucida* Grunow	1.2	0.2	Epipelic
#67	*Nitzschia pseudocommunis* Hustedt	1.6		Epipelic
#68	*Nitzschia sigma* (Kützing) W.Smith	0.8		Epipelic
#69	*Nitzschia valdestriata* Aleem & Hustedt	2.1	3.8	Motile epipsammic
#69[b]	*Nitzschia spp.*	**10.5**	9.0	
#70	*Odontella rhombus* (Ehrenberg) Kützing	0.2		Tychoplankton
#71	*Opephora guenter-grassii* (Witkowski & Lange-Bertalot) Sabbe & Vyverman	0.4	2.7	Stalked
#72	*Opephora horstiana* Witkowski	0.4		Stalked
#73	*Opephora naveana* Le Cohu	0.4	0.2	Stalked
#74	*Paralia sulcata* (Ehrenberg) Cleve	0.4	0.2	Tychoplankton
#75	*Parlibellus berkeleyi* (Kützing) E.J.Cox	0.2	0.5	Tube-dwelling
#76	*Plagiogrammopsis minima* (Salah) Sabbe & A.Witkowski	0.6	3.4	Stalked
#77	*Plagiogrammopsis vanheurckii* (Grunow) Hasle, von Stosch & Syvertsen	4.9	7.0	Tychoplankton
#77[c]	*Plagiogrammopsis spp.*	5.6	**10.4**	
#78	*Planothidium delicatulum* (Kützing) Round & Bukhtiyarova m.1	0.2	0.7	Adnate
#79	*Planothidium delicatulum* (Kützing) Round & Bukhtiyarova m.2	1.2		Adnate
#80	*Planothidium engelbrechtii* (Cholnoky) Round & L.Bukhtiyarova	1.4	1.1	Adnate
#81	*Planothidium sp*.1		0.2	Adnate
#82	*Planothidium sp*.2	0.6		Adnate
#83	*Psammodictyon panduriforme* (W.Gregory) D.G.Mann	0.8		Epipelic
#84	*Rhaphoneis amphiceros* (Ehrenberg) Ehrenberg	0.6	0.2	Stalked
#85	*Surirella atomus* Hustedt	0.2		Epipelic
#86	*Thalassiosira angulata* (W.Gregory) Hasle	0.6	0.5	Plankton
#87	*Thalassiosira guillardii* Hasle		0.2	Plankton
#88	*Thalassiosira minima* Gaarder		1.8	Plankton
#89	*Thalassiosira proschkinae* Makarova	1.0	5.6	Tychoplankton
#90	*Thalassiosira pseudonana* Hasle & Heimdal	3.9	22.1	Tychoplankton
#91	*Thalassiosira sp*.1		0.2	Tychoplankton
#92	*Thalassiosira visurgis* Hustedt	0.2	0.7	Plankton
#92[d]	*Thalassiosira spp.*	5.8	**31.2**	
#93	*Tryblionella apiculata* Gregory	0.8	0.5	Epipelic
